# Spontaneous regression of curve in immature idiopathic scoliosis - does spinal column play a role to balance? An observation with literature review

**DOI:** 10.1186/1749-799X-5-80

**Published:** 2010-11-04

**Authors:** Hitesh N Modi, Seung-Woo Suh, Jae-Hyuk Yang, Jae-Young Hong, KP Venkatesh, Nasir Muzaffar

**Affiliations:** 1 Scoliosis Research Institute, Department of Orthopedics, Korea University Guro Hospital, Seoul, Korea; 2 Rare Disease Institute, Department of Orthopedics, Korea University Guro Hospital, Seoul, Korea

## Abstract

**Background:**

Child with mild scoliosis is always a subject of interest for most orthopaedic surgeons regarding progression. Literature described Hueter-Volkmann theory regarding disc and vertebral wedging, and muscular imbalance for the progression of adolescent idiopathic scoliosis. However, many authors reported spontaneous resolution of curves also without any reason for that and the rate of resolution reported is almost 25%. Purpose of this study was to question the role of paraspinal muscle tuning/balancing mechanism, especially in patients with idiopathic scoliosis with early mild curve, for spontaneous regression or progression as well as changing pattern of curves.

**Methods:**

An observational study of serial radiograms in 169 idiopathic scoliosis children (with minimum follow-up one year) was carried. All children with Cobb angle < 25° and who were diagnosed for the first time were selected. As a sign of immaturity at the time of diagnosis, all children had Risser sign 0. No treatment was given to entire study group. Children were divided in three groups at final follow-up: Group A, B and C as children with regression, no change and progression of their curves, respectively. Additionally changes in the pattern of curve were also noted.

**Results:**

Average age was 9.2 years at first visit and 10.11 years at final follow-up with an average follow-up of 21 months. 32.5% (55/169), 41.4% (70/169) and 26% (44/169) children exhibited regression, no change and progression in their curves, respectively. 46.1% of children (78/169) showed changing pattern of their curves during the follow-up visits before it settled down to final curve. Comparing final fate of curve with side of curve and number of curves it did not show any relationship (p > 0.05) in our study population.

**Conclusion:**

Possible reason for changing patterns could be better explained by the tuning/balancing mechanism of spinal column that makes an effort to balance the spine and result into spontaneous regression or prevent further progression of curve. If this which we called as "tuning/balancing mechanism" fails, curve will ultimately progress.

## Introduction

A major concern of orthopaedic surgeons in managing children with idiopathic scoliosis with a minor curvature is identifying how many and which curve will progress to severe deformities that requires treatment [1-9]. Accurate identification of curves destined to progress requires a clear understanding of the natural history of idiopathic scoliosis. A curve measuring greater than 10°, using the Cobb method, was defined as a structural scoliosis according to the Scoliosis Research Society criteria [10].

Soucacos et al reported 27.4% spontaneous improvement of at least 5° in the curve and 9.5% out of them had complete resolution [11]. Brooks et al reported a 5% incidence of progression in 134 children with a scoliosis of 5° or more, and a spontaneous improvement in 22% [1]. Lonstein and Carlson, in a retrospective review of cases mainly detected through screening at a scoliosis centre, found progression in 23.2% of 727 children with untreated scoliosis [5]. There were numerous factors described causing progression of curve like curve magnitude, skeletal immaturity, sex of patient side of curve and curve pattern etc. However, literature does not explain the reason for spontaneous resolution or regression of curve.

In adolescent idiopathic scoliosis, a larger back muscle volume has been reported at the apex on the convex side of the spine [12,13]. Literature supports the role of paraspinal musculature in progression of curve based on EMG and MRI study [14-16]. Asymmetric myoelectric activity in the convex and concave sides also has been noted [17-21]. Monney et al suggested that asymmetrical spinal muscle activation may not be caused by the curvature itself but may be more primary in the central nervous system [22]. In their study they showed that this muscle imbalance could be corrected by specific exercises that isolate the appropriate musculature. Progress that can be measured can be monitored. Thus there is an ongoing debate suggesting muscle imbalance mechanism causing regression or progression of curve. Weiss suggested that in the younger patients some of the results were not significant or even showed an increase in muscle activity [23]. He attributed this to retarded adaptation of the child's muscles to the changed training conditions [24]. During a muscle training program lasting several weeks, a distinct increase in activity occurs during the first 3 weeks; the activity then drops to the initial value [24,25]. This means that it is quite possible that immature individuals were still in the phase of increased muscle activity. In fact we have observed the changing pattern of curve and Cobb angle especially in younger children with idiopathic scoliosis. They do not show continuous regression or progression rather they show a wavy pattern of their Cobb angle, and sometimes changing the side of their curves. We don't know the reason behind this behaviour of the immature curves.

In present study, we reviewed the curve pattern of immature idiopathic scoliosis patients; and observed different pattern of curve during progression, regression or resolution of curve. Based on our observation we think that tuning or balancing mechanism of spinal column may be an important factor in progression, stabilization or regression of curve, especially in mild degree early curves. We tried to answer our question that in growing spinal muscles or ligaments try to balance the whole spinal column as they get matured and, due to this balancing mechanism mild scoliosis curve shows regression or resolution [24]. Those paraspinal muscles that can't balance enough, scoliosis curve will progress.

## Methods

A retrospective observational study was carried out in 169 idiopathic scoliosis patients (50 male and 119 females) who had regular visits in our outpatient clinic. Average age of patients was 9.2 ± 2.1 years (range, 5~11 years) at the first visit and 10.11 ± 2.8 years (range, 6.1~12.3 years) at final follow-up. Patients who had idiopathic scoliosis, Risser sign 0 at first visit, at least one year regular follow-up, initial curve between 11 and 25 degree and no prior treatment either in form of bracing or manipulation were selected for this study.

**Figure 1:**
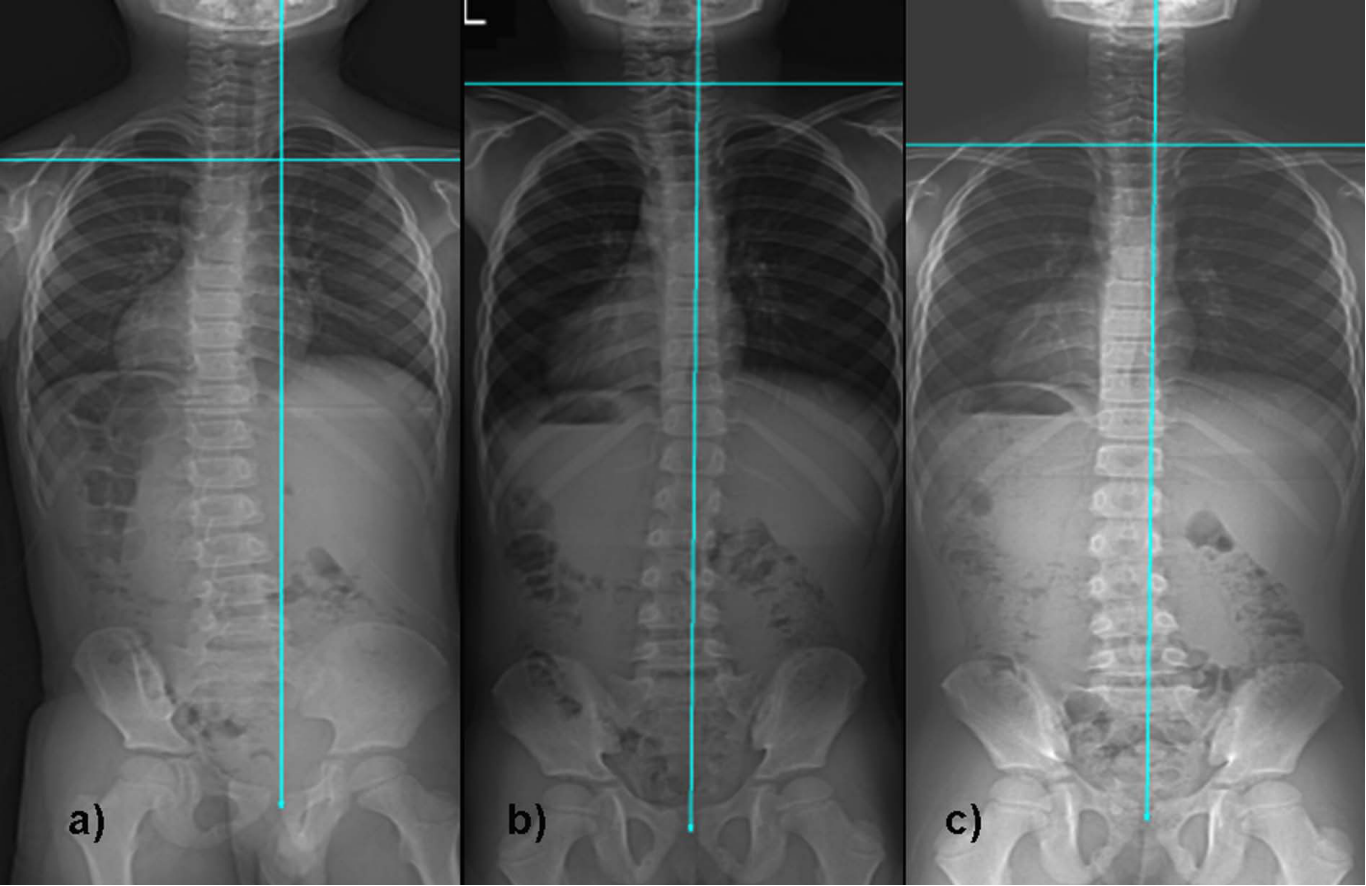
**Serial radiograms of a 6 year old boy with idiopathic scoliosis**. Figure 1a) displayed initial Cobb angle of 15-degrees and curve was on left side; which Figure 1b) became right sided curve with regression of 6-degress after six months; and Figure 1c) again became left side curve after 19 months with Cobb angle of 11-degrees at final follow-up and became stable.

**Figure 2:**
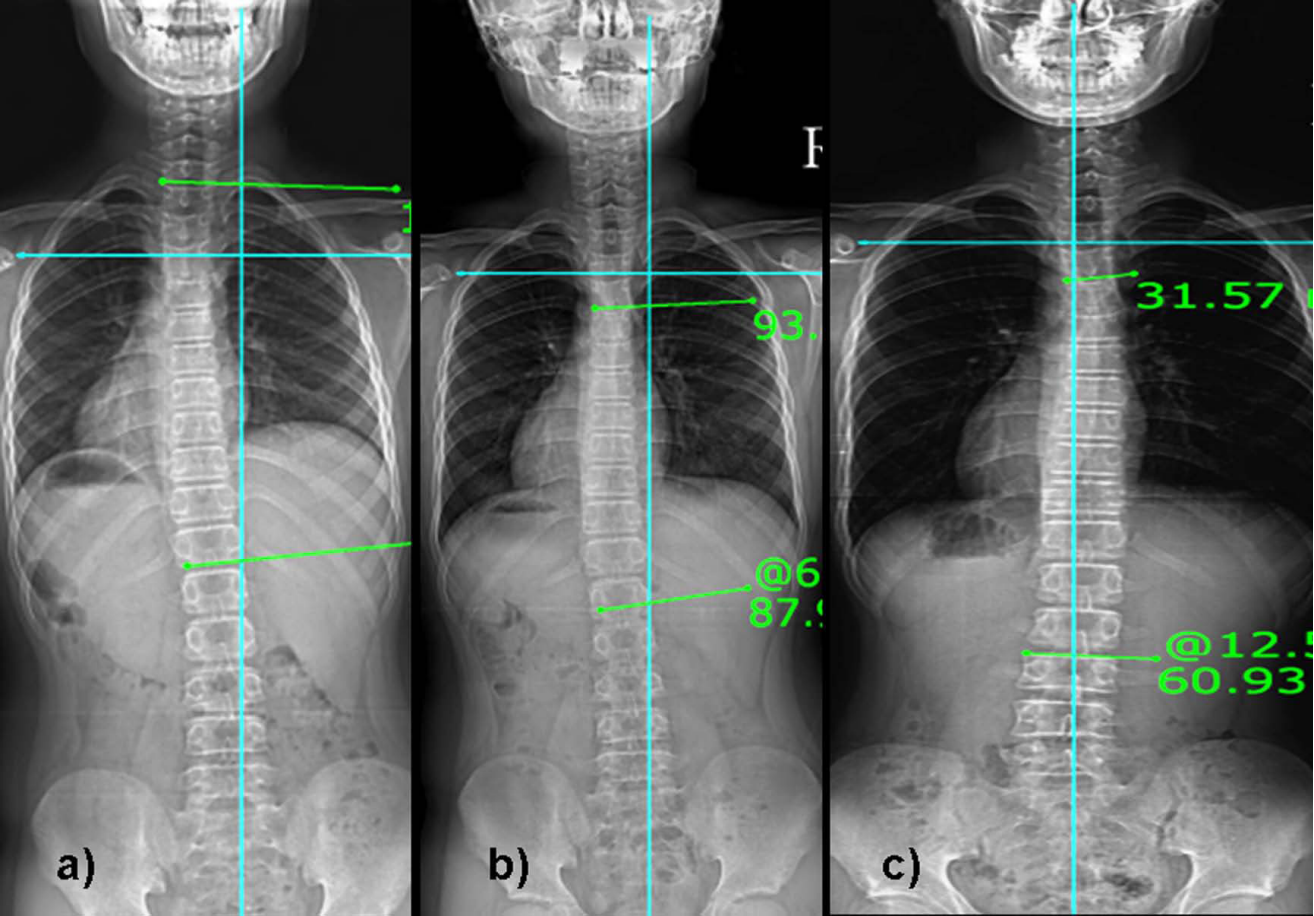
**Serial radiogram of 11 years old male with idiopathic scoliosis**. Figure 2a) showed left sided initial curve with Cobb angle of 8-degrees; which Figure 2b) became 5-degrees after 8 months; and Figure 2c) became right sided after 30 months with Cobb angle of 13-degrees and became stable.

We retrospectively observed serial radiograms for all patients and compared their initial Cobb angle with follow-up and at last follow-up Cobb angle. All children underwent for standing anteroposterior and lateral radiogram of whole spine including both hip joints and full length both lower limb radiograms by a single radiologist. All radiograms were taken on a single X-ray machine to avoid any error. By clinical as well as radiological examination, leg length discrepancy was ruled out in the subject group. Based on our observation, we divided the entire study population into three groups: group A who had regression or resolution of curve for 5° or more at final follow-up; group B who had no change (or difference less than 5°) in Cobb angle at final follow-up; and group C who had progression of curve for more than 5° at final follow-up. We found out the percentage of children in each group from our observation to have an idea of incidence of regression, progression or stabilization of curve. We also tried to find out any change in the curve pattern during each follow-up that might be responsible for regression or progression of curve. Criteria for considering children in changing pattern were the change in side of curve during follow-up or/and wavy pattern of Cobb angle (i.e. sometimes increase and sometimes decrease, they did not display continuous decrease or increasing pattern) (Figure 1 and 2). For the curve showing progression (group C), we considered final follow-up when they required change in the treatment protocol, either in form of bracing or operation.

We also analyzed side of primary curve, number of curves (single or double) and gender of patients in each group to find out any relationship with regression, stabilization or progression of curve. We used Chi-square test to analyze the statistical significance between side of curve, number of curve and gender with curve regression or progression. P value < 0.05 was considered for significance for all tests.

## Results

Average follow-up was 21 ± 9 (range, 12~36) months. Average follow-up in each group with their average age at initial and final follow-up are shown in table 1. Average initial Cobb angle was 15.2° ± 4.6°, 13.9° ± 4.5° and 16.1° ± 4.9° for the group A, B and C, respectively. Average Cobb angle at last follow-up was 6.8° ± 4.3°, 13.6° ± 4.7° and 26° ± 9.3° for the group A, B and C, respectively. Our result showed the incidence rate of 32.5% for children (55/169) who showed regression of curve > 5°, 41.4% for children (70/169) who did not show any change in curve and 26% for children (44/169) who exhibited curve progression > 5° at the latest follow-up. When we observed the serial radiograms of each patient, 46.1% (78/169) exhibited changing pattern in their curves before it settled down to final curve (Figure 1 and 2). This change in the pattern of curve was observed in the children from all three groups; however, the change was more frequently observed in group A and B (p < 0.001).

**Table 1:** Patients' demographics according to group A, B and C.

	All	Group A	Group B	Group C
**Total no of Children (n)**	169	55	70	44
**Male/Female (n)**	50/119	26/29	16/54	8/36
**Average Age (yrs ± SD)**	9.2 ± 2.1	8.11 ± 2.3	9.1 ± 1.11	9.9 ± 1.9
**Average Final Age (yrs ± SD)**	10.11 ± 2.8	10.3 ± 2.9	10.8 ± 2.5	11.11 ± 2.9
**Average Follow-up (yrs ± SD)**	1.9 ± 0.9	1.5 ± 0.4	1.7 ± 0.6	2.2 ± 1.0
**Average Initial Cobb Angle (° ± SD)**	14.9 ± 4.7	15.2 ± 4.6	13.9 ± 4.5	16.1 ± 4.9
**Average Final Cobb Angle (° ± SD)**	14.7 ± 9.6	6.8 ± 4.3	13.6 ± 4.7	26.0 ± 9.3
**Primary Curve side (n)**				
**Right**	122	38	56	28
**Left**	47	17	14	16
**Number of Curves (n)**				
**Single**	87	33	35	19
**Double**	82	22	35	25
**Change in Pattern of Curve (n)**	78	26	44	8

group A who had regression or resolution of curve for 5° or more at final follow-up; group B who had no change (or difference less than 5°) in Cobb angle at final follow-up; and group C who had progression of curve for more than 5° at final follow-up

There were 26 boys and 29 girls in group A; 16 boys and 54 girls in group B; and 8 boys and 36 girls in group C. Comparing fate of curves according to gender it showed significant relationship (p = 0.002, Chi-square test) between gender of patient and curve regression, stability or progression which suggested that boys have greater tendency to stabilize curves while girls have higher tendency for the progression. 38, 56 and 28 curves were right sided and 17, 14 and 16 curves were left sided in group A, B and C, respectively. Comparing the side of curves in each group with final outcome it did not show any relationship (p = 0.14, Chi-square test) between side of curve and regression, stabilization or progression of curve. Similarly there were 33, 35 and 19 children had single curve (thoracic, thoraco-lumbar or lumbar) and 22, 35 and 25 children had double curves (major thoracic and minor lumbar or major lumbar and minor thoracic) in group A, B and C, respectively. Comparing number of curves in each group it also did not exhibit any relationship (p = 0.24, Chi-square test) between number of curves and regression, stabilization or progression of curves.

## Discussion

Results of our study showed that in immature patients who were detected with mild scoliosis for the first time, changing pattern in their curves before displaying regression, stabilization or progression of curves was frequently observed. The incidence rate of changing pattern was noted 46.1%. The possible reason for these changing patterns could be better explained by the tuning/balancing mechanism of paraspinal muscles which try to balance the spine, and result into spontaneous regression or stabilization of curve. If this mechanism which we call as "tuning/balancing mechanism" fails, the curve will ultimately show progression.

Only a subset of curves detected through screening are destined to progress to a point of potential clinical significance. The probability that curves will progress more than 5° can vary from 5% to 90%, depending on the patient's age, sex, and skeletal maturity, and the pattern and magnitude of the curve [5,6,26,27]. Progression is less likely in older children with greater skeletal maturity and with smaller curves [26-28]. Depending on the patient population, between 25% and 75% of curves detected on screening may remain unchanged, and 3-12% of curves may improve [6,28]. The reported probability that curves less than 19° will progress is 10% in girls between age 13 and 15 and 4% in children over this age [5,26]. One study [6] found that the probability was 34% that the curves would progress more than 10°, 18% that they would progress more than 20°, and 8% that they would progress more than 30°. Another study of patients with untreated curves found that 25% ceased progression before reaching 25° and that 12% ceased progression before reaching 29° [28]. However, in the initial stage, especially in skeletally immature children, when curve is identified for the first time, it is difficult to judge whether it will regress, stabilize or progress. In present study we identified 169 children with Risser sign 0 as a sign of skeletal immaturity, and followed them regularly in our outpatient clinic for a minimum period of one year. We observed change in the pattern of curve with their Cobb angle at each follow-up and their fate without any conservative treatment. 32.5%, 41.4% and 26% of the total curve exhibited regression, no change and progression, respectively which can be considered as an incidence rate. However, interestingly, 46% of the total curves showed changing pattern of their curves before they settled in one of these three groups (group A, B or C). These findings aroused curiosity about the significance of changing pattern of curve in the final fate of curve and the reason behind that.

The aetiology of all but idiopathic is self-evident and the progression of deformity is popularly believed to be linked to the mechanical modulation of growth theory [29,30]. It is based on the Hueter-Volkmann principle of differential growth through differential pressure loading on the growth plate [31]. Eular's Law of viscoelastic buckling of a spine in the coronal and transverse planes leading to a lateral bend and axial rotation/torsional buckling, respectively is a mechanical explanation of the forces acting on the vertebral body growth plates as well as the entire spinal column [32,33]. Because scoliosis progresses during the pubescent growth spurt, it is likely that the vertebral body growth plate is a major factor in the development of the scoliosis deformity [34]. The other theory proposed for progression of scoliosis is paraspinal muscle imbalance by several authors. Ford et al [17] suggested that underlying cause of the adolescent idiopathic scoliosis might be the imbalance in the deep muscles at the apex of the curve. They supported the hypothesis of Fidler and Jowett [35] who suggested that increased tonic activity of the deep medial paraspinal muscles, such as multifidus, on one side of the spine and a consequent effect on vertebral growth could be of importance in the aetiology of idiopathic scoliosis. Figueredo and James [36] showed spontaneous resolution of a structural curve, as described in the infantile group of scoliosis, in seven cases (8%) of total 98. Soucacos et al [11] identified the factors important in their association with the natural history of the scoliotic curve regarding sex of the child, curve pattern, and maturity. More specifically, the pattern of the curve was strongly indicative of the risk of progression when considered according to curve direction and sex of the child. Their study group, however, included children from 9 to 15 years of age. In our study, we included children from 5-11 years of age who had Risser sign 0 and skeletally immature. We did not find any relationship between side of curve or number of curve and regression, stabilization or progression of curve; however, boys had higher chances of regression than girls. Additionally, our results proved that there should be possibly other factors responsible, especially in skeletally immature children that might have impact on fate of curve. Since muscles cause movements and maintain tonus, they can be considered to produce skeletal deformities in situations of imbalance [37]. In other words, situations of imbalance of the back muscles may be the only causal factor for scoliosis.

**Figure 3:**
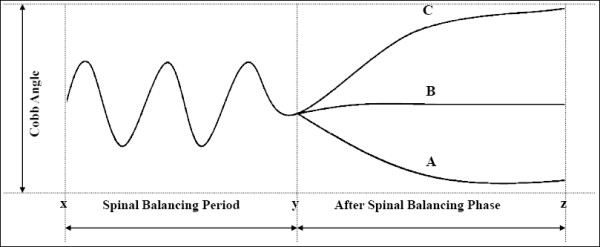
**Explains our proposed hypothesis of tuning/balancing mechanism of spinal column**. Figure shows x is the onset time of scoliosis in growing spine; y is the time when curve will follow one of three pathway (A: regression; B: stabilization and C: progression); and z is the time of change in treatment approach. This figure explains that in growing spine for a short period of time (x-y), there is wavy pattern in Cobb angle that is the period during which spinal column makes an effort to balance the spine. When this effort fails, the curve will follow path C and show the progression; and if it gets balance curve will either stabilize (path B) or regress (path A).

In another experimental study by Schwartzmann and Miles showed that selective muscle imbalance can be produced without muscle excision by the use of inert material to prevent muscle reattachment which will produce lateral curvature [38]. Muscle excision and release which did not produce imbalance resulted in no scoliosis in the animals studied. While Weiss showed decrease in muscular imbalance between convex and concave side with physical rehabilitation program that ultimately reduced the Cobb angle in their subjects [23]. This supported strengthening of the musculature as well as economization of muscle work. However, in the younger patients in his study group, some of the results were not significant or even showed an increase in muscle activity. He attributed this to retarded adaptation of the child's muscles to the changed training conditions [24]. During a muscle training program lasting several weeks, a distinct increase in activity occurred during the first 3 weeks and the activity then dropped to the initial value [24,25]. This proved that immature individuals were still in the phase of increased muscle activity. Thus, based on these literature reviews it is clear that in skeletally immature patients with mild scoliosis paraspinal muscle try to get activated and balance themselves which might be a responsible factor for spontaneous resolution of curve. Once muscles fail to balance and disc or vertebral end plates start showing changes in growth plates, the curve will show progression of curve. We showed that 46% of cases in our study initially exhibited changing pattern in their curve and later on it become stabilized in one pattern. This points out that in skeletally immature children, when curve starts to appear, paraspinal muscles try to balance the spine by their inherent "balancing or tuning mechanism" for a short period of time till it stabilizes into a single pattern. This spinal balancing mechanism might result in a wavy pattern of Cobb angle during the follow-up till it follows one of final path of progression, stabilization or regression (Figure 3). Reviewing literature, we could say that "tuning/balancing mechanism" of paraspinal muscles for the progression, regression or stabilization in immature mild curves; however, the role of spinal ligaments and growth plates cannot be ignored.

The possible criticism for this study might be angle of curve to consider as scoliotic angle. We were concerned about role of spinal column in the developing (immature) curve and that's why we considered those children who had curve more than 10° as per definition of scoliosis. Additionally initial curves of all three groups did not show any significant difference (p = 0.15, ANOVA) in our study. Second criticism might be average follow-up period which is comparatively less with an average of 21 months (minimum 12 months). We would like to clear that actual follow-up period for these patients are longer than that; however, to keep uniform follow-up in all three groups we considered final follow-up when the curves started showing uniform pattern during three follow-up which did not fall in changing pattern category by us. And we did not observe any changes later on as described by us in subsequent follow-up. Cheung et al [15] has established a clear association between both the spinal growth velocity and EMG ratio of the paraspinal muscles and progression of the scoliotic deformity. We believe that in immature children if growth spurt exceeds the paraspinal muscle adaptation rate, the curve will ultimately show progression. And possibly that might be reason that prevalence of scoliosis increases during rapid growth spurt. Role of postural changes [39] can't be ignored in mild scoliotic curves; however, changing pattern from one side of curve to the other side in a same patient on follow-up does not support the role of postural effect. Possibly this could be only explained by our proposed hypothesis of tuning mechanism of paraspinal muscles. We did not do serial EMG study for establishing this hypothesis which may be a lacuna of our study. We believe further work on this issue of tuning mechanism is necessary. Another point might be different level of physical activity of which patient which may affect the nature of curve. However, our study group consisted of only school children and they all were involved in moderate level of physical activity and sports. None of the children was involved in specific sports activity as a professional, and therefore, we don't think that their physical activity might interfere with our results. However, we believe further research would be mandatory especially keeping in mind EMG study and sports level activity. Another criticism might be the age of enrolled children which was 5~11 years, i.e. mixed juvenile and adolescent idiopathic scoliosis which may behave differently. However, our purpose was to see scoliosis in immature spine irrespective of their age. We believe all immature curvature would behave in a same way as published in the literature also. Therefore our results would be valuable to those who are related with scoliosis in their practice.

## Conclusion

Present study shows the possible role of spinal column tuning mechanism in skeletally immature children with mild scoliosis curve for regression, stabilization or progression. If rehabilitation or physical therapy program is applied during this period of immaturity, scoliosis curve might regress with increased activation.

## Competing interests

The authors declare that they have no competing interests. Each author certifies that he has no commercial associations (e.g. consultancies, stock ownership, equity interests, patent/licensing arrangements, etc) that might pose a conflict of interest in connection with the submitted article.

## Authors' contributions

HNM has contributed in conception and design and acquisition of data, analysis and interpretation of data, drafting the manuscript and revising it critically, SWS has contributed in conception and design of data, drafting the manuscript and given the final approval of manuscript, JHY has contributed in acquisition of data, revising the manuscript critically and given the final approval, JYH has contributed in acquisition of data and analysis and interpretation of data; and KPV and NM have contributed in revising the manuscript critically.

All authors read and approved the final manuscript.

## References

[B1] Brooks HL Azen SP Gerberg E Brooks R Chan L Scoliosis: a prospective epidemiological study J Bone Joint Surg [Am] 1975 57 968 972 1194304

[B2] Goldberg CJ Dowling FE Hall JE Emans JB A statistical comparison between natural history of idiopathic scoliosis and brace treatment in skeletally immature adolescent girls Spine 1993 18 902 908 10.1097/00007632-199306000-00015 8316891

[B3] Gross C Graham J Neuwirth M Pugh J Scoliosis and growth: an analysis of the literature Clin Orthop 1983 175 243 250 6839595

[B4] Karol LA Johnston CE Browne RH Madison M Progression of the curve in boys who have idiopathic scoliosis J Bone Joint Surg [Am] 1993 75 1804 1810 8258551 10.2106/00004623-199312000-00010

[B5] Lonstein JE Carlson JM The prediction of curve progression in untreated idiopathic scoliosis during growth J Bone Joint Surg [Am] 1984 66 1061 1071 6480635

[B6] Lonstein JE Natural history and school screening for scoliosis Orthop Clinics North Am 1988 19 227 237 3282198

[B7] Mehta MH The rib-vertebra angle in the early diagnosis between resolving and progressive infantile scoliosis J Bone Joint Surg [Br] 1972 54 230 243 5034823

[B8] Peterson LE Nachemson AL Prediction of progression of the curve in girls who have adolescent idiopathic scoliosis of moderate severity J Bone Joint Surg [Am] 1995 77 823 827 7782354 10.2106/00004623-199506000-00002

[B9] Pin LH Yong L Lin L Hua LK Hui C Hi D Chang B Chang Y Early diagnosis of scoliosis based on school screening J Bone Joint Surg [Am] 1985 67 1202 1205 4055844

[B10] Terminology Committee of the Scoliosis Research Society A glossary of scoliosis terms Spine 1976 1 57 58 10.1097/00007632-197603000-00008

[B11] Soucacos PN Zacharis K Gelalis J Soultanis K Kalos N Beris A Xenakis T Johnson EO Assessment of curve progression in idiopathic scoliosis Eur Spine J 1998 7 270 277 10.1007/s005860050074 9765033 PMC3611270

[B12] Saka K Biomechanical analysis of scoliosis and back muscles using CT evaluation and the finite element method Nippon Seikeigeka Gakkai Zasshi 1987 61 299 310 3624957

[B13] Wood S Magnetic resonance imaging investigation of trunk muscle asymmetry in adolescent idiopathic scoliosis 1996 M.Sc. thesis, Queen's University, Kingston, Ontario, Canada

[B14] Odermatt D Mathieu PA Beausejour M Labelle H Aubin CE Electromyography of scoliotic patients treated with a brace J Orthop Res 2003 21 931 6 10.1016/S0736-0266(03)00038-X 12919883

[B15] Cheung J Veldhuizen AG Halberts JP Sluiter WJ Van Horn JR Geometric and electromyographic assessments in the evaluation of curve progression in idiopathic scoliosis Spine 2006 31 322 29 10.1097/01.brs.0000197155.68983.d8 16449906

[B16] Zoabli G Mathieu PA Aubin CE Back muscles biometry in adolescent idiopathic scoliosis The Spine Journal 2007 7 338 344 10.1016/j.spinee.2006.04.001 17482118

[B17] Ford DM Bagnall KM McFadden KD Greenhill BJ Raso VJ Paraspinal muscle imbalance in adolescent idiopathic scoliosis Spine 1984 9 373 76 10.1097/00007632-198405000-00008 6474252

[B18] Reuber M Schultz A McNeill T Spencer D Trunk muscle myoelectric activities in idiopathic scoliosis Spine 1983 8 447 56 10.1097/00007632-198307000-00002 6228013

[B19] Riddle HFV Roaf R Muscle imbalance in the causation of scoliosis Lancet 1955 i 1245 47 10.1016/S0140-6736(55)91020-5 14382561

[B20] Zetterberg C Bjork R Ortengren R Andersson GBJ Electromyography of the paravertebral muscles in idiopathic scoliosis Acta Orthop Scand 1984 55 304 9 10.3109/17453678408992362 6741480

[B21] Zuk T The role of spinal and abdominal muscles in the pathogenesis of scoliosis J Bone Joint Surg [Br] 1962 44 102 5

[B22] Mooney V Gulick J Pozos R A Preliminary Report on the Effect of Measured Strength Training in Adolescent Idiopathic Scoliosis Journal of Spinal Disorders 2000 13 2 102 107 10.1097/00002517-200004000-00002 10780683

[B23] Weiss HR Imbalance of electromyographic activity and physical rehabilitation of patients with idiopathic scoliosis Eur Spine J 1993 1 240 243 10.1007/BF00298367 20054925

[B24] Basmajian JV De Luca CJ Muscles alive, their function revealed by electromyography 1985 Williams & Wilkins, Baltimore

[B25] Smidt GL Fapta PT Blanpied PR Exploration of mechanical and electromyographic responses of trunk muscles to high-intensity resistive exercise Spine 1989 14 815 830 10.1097/00007632-198908000-00008 2528814

[B26] Nachemson A Lonstein JE Weinstein SL Report of the prevalence and natural history committee 1982 Park Ridge, IL: Natural History Committee of Scoliosis Research Society

[B27] Bunnell WP The natural history of idiopathic scoliosis before skeletal maturity Spine 1986 11 773 776 10.1097/00007632-198610000-00003 3810290

[B28] Willner S Uden A A prospective prevalence study of scoliosis in southern Sweden Acta Orthop Scand 1982 53 233 237 10.3109/17453678208992208 7136569

[B29] Stokes I Spence H Aronsson D Kilmer N Mechanical modulation of vertebral body growth. Implications for scoliosis progression Spine 1996 21 1162 1167 10.1097/00007632-199605150-00007 8727190

[B30] Stokes I Mente P Iatridis J Farnum C Aronsson D Enlargement of growth plate chondrocytes modulated by sustained mechanical loading J Bone Joint Surg 2002 84A 1842 1848 10.2106/00004623-200210000-00016 12377917

[B31] Mehlman C Araghi A Roy D Hyphenated history: the Hueter- Volkmann Law. History of Orthopedics Am J Orthop 1997 9402217

[B32] Asher M Burton D A concept of idiopathic scoliosis deformities an imperfect torsion(s) Clin Orthop 1999 364 11 25 10.1097/00003086-199907000-00003 10416387

[B33] Goto M Kawakami N Azegami H Matsuyama Y Takeuchi K Sasaoka R Buckling and bone modeling as factors in the development of idiopathic scoliosis Spine 2003 28 364 370 10.1097/00007632-200302150-00010 12590211

[B34] Urban J Stokes IAF Regulation of spinal growth and remodeling Research into Spinal Deformities 1999 2 Amsterdam: IOS Press 12 17

[B35] Fidler MW Jowett RL Muscle imbalance in the aetiology of scoliosis J Bone Joint Surg [Br] 1976 58 2 200 201 932082 10.1302/0301-620X.58B2.932082

[B36] Figueredo UM James JIP Juvenile idiopathic scoliosis J Bone Joint Surg [Br] 1981 63 1 61 66 7204475 10.1302/0301-620X.63B1.7204475

[B37] Chagas JCM Schimidt B Puerta EB Oliveira CEAS Freita AA Estudo histoquímico dos músculos rotadores do dorso em pacientes com escoliose idiopática do adolescente Rev Bras Ortop 1998 33 2 111 8

[B38] Schwartzmann JR Miles M Experimental production of scoliosis in rats and mice J Bone Joint Surg Am 1945 27 59 69

[B39] Stehbens WE Pathogenesis of idiopathic scoliosis revisited Exp Mol Pathol 2003 74 49 60 10.1016/S0014-4800(03)80008-4 12645632

